# Tofacitinib in the treatment of primary Sjögren’s syndrome-associated interstitial lung disease: study protocol for a prospective, randomized, controlled and open-label trial

**DOI:** 10.1186/s12890-023-02774-0

**Published:** 2023-11-25

**Authors:** Ronglin Gao, Jincheng Pu, Yanqing Wang, Zhenzhen Wu, Yuanyuan Liang, Jiamin Song, Shengnan Pan, Fang Han, Lufei Yang, Xianghuai Xu, Jianping Tang, Xuan Wang

**Affiliations:** 1 Department of Rheumatology and Immunology, Tongji Hospital, School of Medicine, Tongji University, No. 389 Xincun Road, Shanghai, 200065 China; 2 Department of Pulmonary and Critical Care Medicine, Tongji Hospital, School of Medicine, Tongji University, No. 389 Xincun Road, Shanghai, 200065 China

**Keywords:** Tofacitinib, Primary Sjögren’s syndrome, Interstitial lung disease, Cyclophosphamide, Azathioprine

## Abstract

**Introduction:**

Tofacitinib, a selective inhibitor of JAK1 and/or JAK3, is considered to alleviate the pulmonary condition of primary Sjögren’s syndrome (pSS)-associated interstitial lung disease (ILD) through its anti-inflammatory and antifibrotic effects.

**Methods and analysis:**

This is a single-center, prospective, randomized, open-label trial. The trial will compare a 52-week course of oral tofacitinib with traditional therapy cyclophosphamide (CYC) combined with azathioprine (AZA) in the treatment of pSS-ILD. A total of 120 patients will be randomly assigned into two treatment groups with a 1:1 ratio and followed for 52 weeks from the first dose. The primary endpoint of the study is the increase of forced vital capacity (FVC) at 52 weeks. Secondary endpoints include high-resolution computed tomography (HRCT), diffusion capacity for carbon monoxide of the lung (DLCO), the Mahler dyspnea index, the health-related quality of life (HARQoL) score, the cough symptom score, EULAR Sjögren's syndrome disease activity index (ESSDAI), and safety.

**Discussion:**

This study will be the first randomized controlled trial to investigate tofacitinib compared to the traditional regimen of CYC in combination with AZA in the treatment of pSS-ILD, which will provide data on efficacy and safety and further elucidate the role of the JAK-STAT signaling pathway in the development of pSS-ILD.

**Ethics and dissemination:**

Before starting the experiment, the research proposal, informed consent (ICF) and relevant documents in accordance with the ethical principles of the Helsinki Declaration and the relevant requirements of the local GCP rules for ethical approval shall be submitted to the ethics committee of the hospital. The ethical approval of this study is reviewed by the Ethics Committee of Tongji Hospital and the ethical approval number is 2021-LCYJ-007. When the experiment is completed, the results will also be disseminated to patients and the public through publishing papers in international medical journals.

**Trial registration:**

The study was registered on the Chinese Clinical Trial Registry, www.chictr.org.cn; ID ChiCTR2000031389.

**Supplementary Information:**

The online version contains supplementary material available at 10.1186/s12890-023-02774-0.

## Background

Primary Sjögren’s syndrome (pSS) is a chronic inflammatory autoimmune disease characterized by lymphocytes infiltration and proliferation of exocrine gland and small vasculitis. In the early clinical stage, the function of salivary glands and lacrimal glands is impaired, multiple organs are gradually involved in the later, especially the lungs. Interstitial lung disease (ILD) is a common injury type of lungs in pSS patients, with an incidence of up to 13%, which results in poor life quality and high mortality [1, 2]. For patients with progressive or severe lung disease, first-line therapy is usually based on glucocorticoids, alone or in combination with immunosuppressive drugs. The usual initial dose of glucocorticoids is 0.5–1 mg/kg prednisone per day, depending on the severity of ILD and the classical regimen is combined with immunosuppressive agents such as cyclophosphamide (CYC), mycophenolate mofetil (MMF), or azathioprine (AZA) [3], however, due to the toxicity of CYC, high incidence of serious adverse reactions, poor tolerance and other problems, the treatment receives certain limitations. In recent years, biologic agents such as rituximab have been shown a safe and effective alternative to CYC for the treatment of pulmonary manifestations in connective tissue disease (CTD) [4]. Some patients with obvious fibrosis progression need to be treated with anti-fibrosis drugs (nintedanib, etc.), which united with immunosuppressants can have a favorable effect on the pulmonary function and overall tolerance of patients, but only slow the progression, and large-scale sample exploratory research is needed to confirm [5]. Therefore, janus kinases (JAKs) inhibitors with anti-inflammatory and antifibrotic effects would be a new choice for CTD-ILD treatment [6–8].

JAKs play a key role in many cytokines signaling pathways: primarily including type I interferons (IFNs), interleukin-6 (IL-6), which mediate changes in cell activation, proliferation, and survival [9]. Dysregulation of JAK- signal transductor and activator transcription (STAT) pathway has been linked to a variety of inflammatory responses and immune disorders, such as rheumatoid arthritis (RA) and systemic lupus erythematosus (SLE) [10, 11]. Related factors (such as IL-6 and IL-2) play an important role in the pathogenesis of GPA by activating the JAK-STAT signaling pathway, leading to dysfunction of CD4 + T cells [12]. In Behcet's disease, activation of the JAK-STAT pathway has also been observed in activated monocytes and CD4 + T cells, in which highly expressed STAT1 and STAT2 are involved [13]. In recent years, there has been growing interest in modulating the JAK-STAT pathway for the treatment of CTD [14, 15], and JAK inhibitor has been shown a new therapeutic target.

Tofacitinib is a novel oral inhibitor with functional selective inhibition of JAK1 and/or JAK3. It can act on the synovial JAK-STAT signaling pathway of RA, reducing the expression of matrix metalloproteinases (MMP) and interferon-regulated genes in synovial cells [16]. Researchers have also found abnormal upregulation of IFN signals and different levels of IL-6 expression in inflammatory cells and muscle fibers in dermatomyositis, tofacitinib may also alleviate the active state of dermatomyositis by significantly down-regulating the transduction pathway [17, 18]. A single-center study confirmed that tofacitinib partially improved the signs and symptoms of arthritis and the appearance of rash in SLE patients [19]. Other studies have also concluded that tofacitinib improves clinical symptoms and inflammatory parameters in AAV patients without organ involvement, while saving the dose of glucocorticoids [20]. However, the clinical application of JAK inhibitors in CTD-ILD is still being explored. JAK-STAT signaling pathway is also a key process for macrophage polarization [21]. In a mouse model of systemic sclerosis, JAK inhibitors also inhibit proinflammatory M1 macrophages and profibrotic M2 in the lungs and skin [22, 23]. Tofacitinib also significantly inhibited the development of RA-ILD mice. The mechanism may be related to the inhibition of CD4 + T cell proliferation and Th17 cell differentiation by increasing myeloid-derived suppressor cells (MDSC) in inflamed lungs, which is a potential treatment option for RA-ILD [24].

These studies suggest that tofacitinib has not only anti-inflammatory but also antifibrotic effects, highlighting its advantages in the treatment of ILD, especially CTD-ILD. In addition to pSS-ILD, clinical studies have confirmed the potential of tofacitinib in the treatment of CTD-ILD. A retrospective clinical study found that tofacitinib mitigated the degree of lung fibro degeneration through assessing by high resolution computed tomography (HRCT) in patients with RA-ILD [25]. According to the Chinese study, tofacitinib significantly reduces the mortality of patients with anti-MDA5-positive dermatomyositis-associated ILD, which can not only increase the 6-month survival rate, but considerably improve the results of forced vital capacity (FVC), diffusion capacity for carbon monoxide of the lung (DLCO) and HRCT, with the lower risk of adverse events [26]. Many foreign scholars also confirmed that tofacitinib can make progress in the proinflammatory and profibrotic effects of T-cell source in dermatomyositis patients with ILD, especially in the rapidly progressive ILD [27].

For pSS, existing research on the role of tofacitinib is also deepening. Barrera MJ et al. found that tofacitinib not only reduced the inflammatory response in LSG epithelial cells of pSS patients, but also inhibited the reduction of autophagy [28]. Targeting the downstream signals of interferon and other pro-inflammatory cytokines such as IL-6 and IL-17 through the JAK pathway may also benefit the lung involvement of pSS [6]. In addition, filgotinib, an inhibitor of JAK1, has been shown to improve salivary gland function and B cell activity in animal models of pSS [29]. Related clinical trials are also underway, suggesting that filgotinib may be suitable for pSS patients with high EULAR Sjögren's syndrome disease activity index (ESSDAI) and is generally well tolerated by patients [30]. Evidence from these studies shows new hope that JAK inhibitors can be used to treat pSS.

We considered whether tofacitinib could play both anti-fibrosis and anti-inflammatory effects, which would be a new opportunity for the treatment of pSS-ILD. To our knowledge, no studies have evaluated the efficacy and associated adverse events of tofacitinib with clinical pSS-ILD patients. In this study, the long-term efficacy and safety of tofacitinib in the treatment of pSS-ILD will be studied by comparing the classical treatment of CYC followed by AZA.

### Aims

- To evaluate the efficacy of tofacitinib in the treatment of pSS-ILD after 52 weeks of administration, which will be noninferior to CYC with AZA.
- To investigate the suitable population, clinical efficacy indicators, effects on cellular and humoral immunity, and possible clinical side effects of tofacitinib for providing a basis for clinical application in pSS-ILD in the future.

## Methods

### Design and setting

**Fig. 1:**
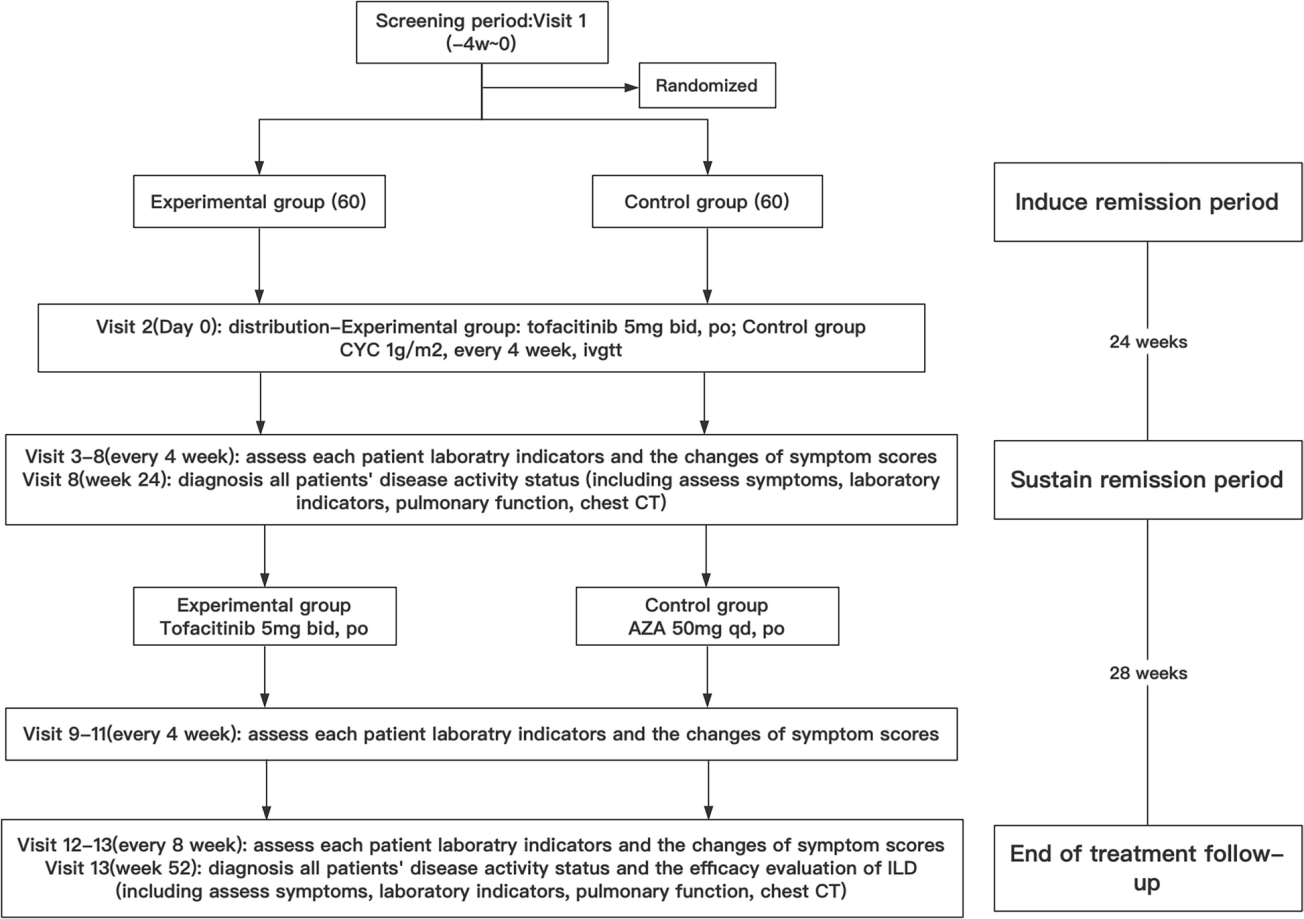
Flowchart of study design

The study is a single-center, prospective, randomized, controlled and open-label trial, designed to identify the impact of tofacitinib in treating active pSS-ILD compared with CYC sequential AZA treatment. The study will recruit subjects from Shanghai Tongji Hospital. The schedule of events for the enrolment, interventions and assessments of participants is shown in Fig. 1 and specific follow-up is shown in Table 1. The protocol is reported according to the Standard Protocol Items: Recommendations for Interventional Trials (SPIRIT) guidelines. The SPIRIT checklist is provided in Additional file 1.

**Table 1:** Outline of planned visit

	**STUDY PERIOD**							**Follow-up of patients with early termination**
	**Screening Visit**	**Randomize**	**Treatment Visits**					
TIMEPOINT**	**Visit 1** ***-4w* ~ *0***		**Visit 2** **Day 0**	**Visit 3–7** **Week 4–20**	**Visit 8** **Week 24**	**Visit 9–12** **Week 28–44**	**Visit 13** **Week 52**	
**Eligibility screen**	✔							✔
**Informed consent**	✔							✔
**Adverse event check**				✔	✔	✔	✔	✔
**Study drug**			✔	✔	✔	✔	✔	✔
**Concomitant medication**			✔	✔	✔	✔	✔	✔
**ASSESSMENTS:**								
**Physical exam**	✔			✔	✔	✔	✔	✔
**ESSDAI score**	✔				✔		✔	✔
**Cough symptom score**			✔	✔	✔	✔	✔	✔
**HARQoL score**			✔	✔	✔	✔	✔	✔
**Mahler dyspnea index**	✔			✔	✔	✔	✔	✔
**6-MWT**			✔	✔	✔	✔	✔	✔
**Routine laboratory examination**	✔			✔	✔	✔	✔	✔
**C3, C4, IgA, IgG, IgM, IgG4**			✔	✔	✔	✔	✔	✔
**Autoimmune antibody spectrum**			✔		✔		✔	✔
**CD series cells**			✔		✔		✔	✔
**Arterial blood gas**	✔			✔	✔	✔	✔	✔
**Pulmonary function**	✔				✔		✔	✔
**Chest CT**	✔				✔		✔	✔

*Abbreviation*: *ESSDAI* EULAR Sjögren's syndrome disease activity index, *HARQoL* Health-related quality of life, *6-MWT* 6-min walk test, *C3* Complement 3, *C4* Complement 4, *IgA* immunoglobulin A, *IgG* Immunoglobulin G, *IgM* Immunoglobulin M, *IgG4* Immunoglobulin G4, *CT* Computed tomography

### Patient selection

At present, there is no accepted diagnostic standard for CTD- ILD, due to the professionalism of HRCT reading and pulmonary function report interpretation, rheumatologists, respiratory physicians and radiologists are involved in the diagnostic process. To be eligible for inclusion participants will meet the following criteria:

- 18 years ≤ age ≤ 75 years;
- Patients are eligible for the pSS classification criteria for 2002/2016 [31, 32];
- Patients are eligible for the ILD classification criteria [33];
- Exertive dyspnea is present and the Mahler dyspnea modified index task grade 2 is achieved;
- Lung function: FVC accounted for ≥ 45% of predicted values, DLCO ≥ 30% of predicted values, forced expiratory volume in one second (FEV1) /FVC > 65%;
- If glucocorticoids are used at the time of screening, the dose of prednisone acetate is less than 30mg/d (or equivalent amounts of other types);
- Patients who haven’t used immunosuppressive agents (including but not limited to CYC, cyclosporin A (CsA), AZA, tacrolimus (FK-506), methotrexate (MTX), leflunomide, MMF, etc.) or have been stopped for ≥ 3 months at the time of screening; when hydroxychloroquine (HCQ) was used, the dose of HCQ was stable for 3 months or more;
- Patients who are not using biologics (including but not limited to rituximab, infliximab, adalimumab, etanercept, etc.) at the time of screening or have stopped medication for ≥ 3 months;
- Women of reproductive age must have a negative urine pregnancy test. From the beginning of the screening period until the last use, both fertile women and men must voluntarily use a recognized effective contraception;
- Able to read, understand and give written informed consent.

### Exclusion criteria

Subjects meeting any of the following criteria will not be eligible to participate:

- Patients with acute exacerbation of interstitial pneumonitis (AEIP);
- Respiratory failure suggested by arterial blood gas analysis;
- Other lung lesions other than ILD are evaluated by the following criteria:

a: Patients with moderate to severe pulmonary hypertension which is assessed by rheumatological experts need special treatment;

b: Smoking in the past 6 months or now still a smoker;

c: Patients with other severe clinical manifestations of pulmonary disease, such as pulmonary mass or active pulmonary infection;

d: Patients with severe lung disease other than ILD indicated by lung biopsy, alveolar lavage, or HRCT;

- Severe heart, liver, kidney and other important organs lesions;
- The active infection is aggravated by glucocorticoid and immunosuppressive therapy;
- Positive for hepatitis B virus surface antigen or hepatitis C antibody;
- Pregnant and lactating women, or childbearing age can’t ensure effective contraception;
- Tofacitinib, glucocorticoids, CYC, AZA allergy or intolerance.

### Sample size estimate

**Fig. 2:**
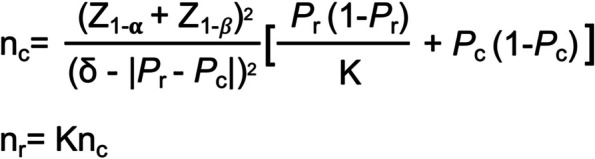
Sample size calculation formula

The sample size will be calculated according to the following formula. According to the previous data, the effective *P*_r_ of the experimental group was 0.8, and the effective *P*_c_ of the control group was 0.7. The non-inferiority margin value δ is set as -0.1. With K value of 1, this trial is a non-inferior efficacy study with unilateral detection. They will be randomly divided into tofacitinib group or control group in a ratio of 1:1. Finally, the sample size is calculated as 57 according to the following formula (Fig. 2). Assuming a 5% shedding rate, we need to include about 60 subjects in each group.

### Randomization, sequence generation and allocation concealment

SPSS statistical software is used to generate a random coding table with serial numbers (01 ~ 120) for random assignment of experimental drugs. Patients will be assigned by a random code in strict order and treated with the experimental drug assigned by the code, when they meet the randomization criteria during the screening period. This process will be performed independently by statistical professionals using SPSS software who aren’t involved in subsequent interventions and follow-up with the patient.

### Interventions

**Fig. 3:**
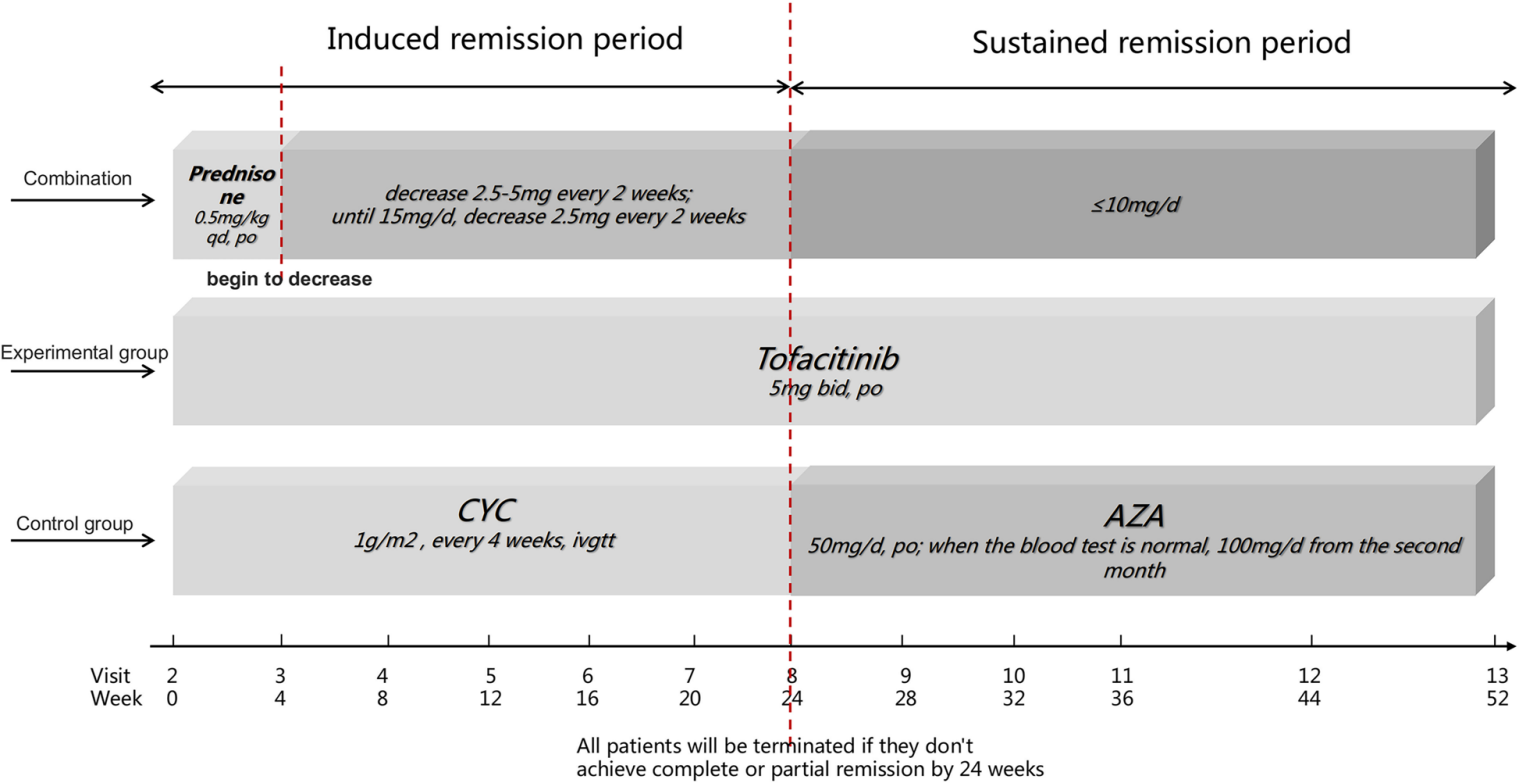
The administration process between the experimental group and control group. Abbreviations: CYC: cyclophosphamide; AZA: azathioprine

In the treatment group (tofacitinib group), 60 pSS patients will receive tofacitinib 5mg twice daily until 52 weeks. Control group (conventional treatment group) containing 60 pSS patients receives conventional classical CYC sequential AZA treatment. Concretely speaking, the control group will receive CYC induction therapy which means intravenous infusion of 1g/m^2^ every 4 weeks. The patients will be terminated in trial with continued progression of pulmonary lesions and worsening dyspnea by 24 weeks. Patients who achieve partial or complete remission after CYC induction therapy will continue to be administrated with oral AZA maintenance therapy. Oral administration is given with an initial dose of 50 mg/day and will increase to 100mg/day in the second month if there is normality in blood examination weekly. The specific administration process is shown in Fig. 3.

### Reduction or discontinuation

Discontinuation of the trial drug during the trial should be avoided. However, if one of the following conditions occurs, the researcher can consider reducing the dose, suspending or discontinuing it:

- The serious advent events (SAE) which has a certain relationship with the trial drug occurs;
- The allergic reaction of experimental drug occurs, and symptomatic treatment can’t effectively alleviate;
- Other concurrent/newly developed diseases which may be aggravated by the experimental drugs;
- The laboratory indicators related to safety are obviously abnormal;
- The researcher considers other circumstances in which reduction, suspension, or discontinuation is warranted.

### Concomitant medication

- Glucocorticoid: All patients will be treated with glucocorticoid. Dose (all are calculated as prednisone): 1–4 weeks, 0.5mg/kg; starting from week 5, reducing 2.5-5mg every 2 weeks; after reducing to 15mg/d, reducing 2.5mg every 2 weeks. The dose of prednisone should not exceed 10mg/d by the end of the induced remission period and the maintenance period. If the patient develops severe extrapulmonary symptoms during the follow-up period, the dose of hormone can be increased by 1mg/kg*d, but no more than 2 weeks.
- Antimalarial drugs, including HCQ and chloroquine, may be used in combination.
- Immunosuppressants other than CYC and AZA, including MMF, CsA, TC, MTX, leflunomide and so on, should not be used in combination.

### Efficacy measurement procedures

The outcome measures include cough symptom score, the health-related quality of life (HARQoL) score, the Mahler dyspnea index, 6-min walk test (6MWT), ESSDAI, pulmonary function, HRCT, laboratory routine and immune markers. ILD activity status (including HRCT, pulmonary function, symptoms) will be performed at baseline and 52-week treatments to determine the efficacy of treating ILD. Any side effects associated medicines will be recorded. The assessment of questionnaire will be finished by the subjects in cooperation with the researcher. Biological samples required for this study will be collected, processed and analyzed in accordance with the prescribed procedures, and all indicators will be standardized tested in the clinical laboratory of the Tongji hospital. All techniques will be carried out in accordance with the methods specified in the relevant international and national guidelines.

### Discontinuation or withdrawal of study subjects

Subjects may voluntarily withdraw from the trial at any time for any reason, and the investigator may discontinue any subject's participation in the trial for various reasons, including safety concerns or protocol violations. Subjects shall withdraw from the trial or be deemed to withdraw from the trial under any of the following conditions:

Subjects are not willing to continue:

- Due to poor or ineffective efficacy, the subjects voluntarily stop taking the drug and refuse to continue participating in the trial;
- Loss to follow-up (if the cause is traffic accident, death, fracture and other accidents, the investigators should timely follow-up and judge the causal relationship with the test drug);
- The subjects withdraw the informed consent;

SAE, major adverse events or severe allergic reactions, etc.:

- The investigator and/or the monitor consider the subject inappropriate to continue using the trial drug based on safety and ethical considerations due to significant abnormalities in laboratory indicators related to safety, or SAE, or severe allergic reactions;
- Deterioration of the original disease or complicated/new other serious diseases, such as tuberculosis, tumor, the researchers judge that it is not appropriate to continue to participate in the trial;

Violation of test protocol:

- Serious protocol violations due to the investigator, such as non-inclusion criteria and/or exclusion criteria;
- The subjects don’t sign the informed consent;
- The subjects fail to complete the required examination on time, resulting in failure to perform the evaluation;
- Unintended pregnancy during the trial.

### Statistical analysis

SPSS software will be used for statistical analysis. Single-tailed tests are used for all statistical tests. *P* value < 0.05 is considered statistically significant with a confidence interval of 95%. The non-inferiority margin value δ is set as -0.1. Quantitative variables will be described by means ± standard deviation or median (interquartile spacing) using t-test and nonparametric test to compare the differences between groups. Categorical variables will be statistically described by frequency (constituent ratio), and the changes before and after treatment calculated by chi-square test or Fisher exact test.

### Patient and public involvement statement

Patients will participate in and terminate the study voluntarily. They are not involved in the design and dissemination plans of the study. Some of the outcome indicators will be patient-reported in collaboration with the investigator, including cough symptoms, HARQoL score and others, and timely feedback on safety (adverse effects) during the treatment.

## Outcome measures

### Primary outcome measure

Evaluating the changes of FVC in pSS-ILD patients at 52-week follow-up.

### Secondary outcome measures

- Evaluating the changes of pulmonary interstitial lesions: the change of chest HRCT;
- Evaluating the changes of DLCO in pulmonary function;
- Measure of the patients' cardiopulmonary function: 6MWT;
- Assessment for improvement in respiratory symptoms: the changes of the Mahler dyspnea index and cough symptom score;
- Subjective valuation of overall health status: changes of the HARQoL score;
- Assessment of pSS disease activity will be undertaken using ESSDAI.

### Other outcome indicators

- Dosage of hormone: the reduction of glucocorticoid dosage and its speed; the proportion of doses ≤ 7.5mg/d maintained for more than 90 days;
- Serum immunoglobulin A (IgA), IgM, IgG, IgG4, complement 3 (C3), C4, rheumatoid factor (RF), IL-1β, IL-2R, IL-6, IL-8, tumor necrosis factor-α (TNF-α), antibody profiles;
- Peripheral blood immune cell profile: CD3, CD4, CD8, CD19, CD16 + CD56 + cell, CD4/CD8;
- Routine laboratory indications: blood routine, liver/renal function, arterial blood gas analysis and so on.

### Efficacy evaluation criteria of ILD

Guidelines were used to assess the response to treatment and clinical course of ILD by taking into account a combination of factors [34].

Effective: ILD has completely improved (at the same time meeting the following requirements):

- Chest CT: the interstitial lung lesions are reduced by more than 10% (score) and without new lesions;
- Pulmonary function assessment: increase of ≥ 10% in FVC and/or ≥ 15% in DLCO;
- Symptoms: increase in cough symptom score ≥ 50% and increase in the Mahler dyspnea index ≥ 10%.

Partially effective: ILD is stable (at the same time meeting the following requirements)

- Chest CT: -10% < increase in the changes of HRCT < 10% (score), no new lesions;
- Pulmonary function assessment: -10% < increase in FVC < 10%; -15% < increase in DLCO < 15%;
- Symptoms: -50% < increase in cough symptom score < 50%; -10% < increase in the Mahler dyspnea index < 10%.

Ineffective: ILD is progressing (meeting any of the following conditions, excluding infection):

- There is an acute exacerbation of ILD;
- Chest CT: increase in the changes of HRCT ≤ -10% (score), or new lesions;
- Pulmonary function assessment: increase in FVC ≤ -10%; increase in DLCO ≤ -15%;
- Symptoms: increase in cough symptom score ≤ -50%; increase in the Mahler dyspnea index ≤ -10%.

### Assessment of adherence

Subjects should return the experimental drug after the treatment period. To judge the compliance of subjects, medication compliance should be 80% ~ 120% in general.

Adherence will be assessed as actual medication dose/theoretical medication dose × 100%.

$$Actual\, dose = total \,amount \,of \,medication \,dispensed - (total \,amount \,of \,remaining \,return \, \text{+} \, total \,amount \,of \,lost)$$

$$Theoretical \,dose = individual \,dose \times \,number.$$

Good adherence: 80% to 120%; poor adherence: 120%.

### Data management and protocol amendments

This study will use Excel software input to save the subject information. Data entry personnel have the rights of data entry, modification and question; the researchers have the right to modify, browse, question and review data; finally, supervisors have the rights to browse, send/close doubts, freeze and lock data. Tongji Hospital has Data Security and Monitoring Committee (DSMB). Data security and monitoring reports will be submitted quarterly to the DSMB. The applicant shall submit a progress report to the ethics committee for trial review one month before the due date in accordance with the annual follow-up review frequency. If the principal investigator is changed during the study, any changes to the clinical study protocol, informed consent, recruitment materials, etc., shall be submitted to the ethics committee for amendment review.

### Safety assessment

Researchers should evaluate the severity of AE based on medical judgment, not directly on the subject's experience. According to the following criteria, AE severity can be divided into mild, moderate and severe:

- Mild: transient, mild symptoms which don’t interfere with daily life and require special measures or treatment.
- Moderate: mild impact on daily life and actions, requiring measures or treatments if necessary.
- Severe: severely affecting daily life and activities, requiring special measures or treatment, and hospitalization if necessary.

Start collecting AE after subjects sign ICF. All AEs must be recorded in the appropriate section of the case report form (CRF), regardless of whether the AE is related to the drug being tested. If AE occurs, researchers can decide whether to take medical measures according to the condition. In the presence of SAE, the investigator must immediately conduct proper treatment or rescue treatment to protect the safety of the subject. In the course of this study, if any SAE occurs, the researcher should report it to the drug regulatory department and the ethics committee of the research center within 24 h after learning about the SAE.

### Ancillary and post-trial care

The sponsor will pay the reasonable transportation costs incurred by participation in the study, and there will be no additional expenses for subjects. If the subjects suffer any injury during the study or any injury that is causally related to the trial drug used, the sponsor will bear the medical expenses and provide subjects with the corresponding economic compensation in accordance with relevant national laws and regulations. Even if subjects have signed this informed consent, they still retain all of legal rights and terminate the study at any time.

## Discussion

ILD is one of the most common and serious pulmonary complications of pSS, which poses a great threat to the long-term survival of patients. Due to the lack of observation and confirmation of a large number of clinical trials, pSS-ILD is still mainly empirical treatment, that’s means more effective treatments are needed. At present, the application of JAK inhibitors in autoimmune diseases continues to mature, and the basic experiment and clinical efficacy of RA and dermatomyositis with ILD have been confirmed by domestic and foreign scholars. Meanwhile, this study will be the first randomized controlled clinical trial to study tofacitinib in the treatment of pSS-ILD, which can provide data on the efficacy and safety. It may bring new hope for the future treatment of CTD-ILD by JAK inhibitors, and further clarify the role of JAK-STAT signaling pathway in the development of CTD-ILD.

### Supplementary Information

**Additional file 1.**

**Additional file 2.**

## Data Availability

Not applicable.

## Abbreviations

pSS: Primary Sjögren’s syndrome
ILD: Interstitial lung disease
CYC: Cyclophosphamide
AZA: Azathioprine
MMF: Mycophenolate mofetil
CTD: Connective tissue disease
JAKs: Janus kinases
IFN: Type I interferon
IL-6: Interleukin-6
STAT: Signal transductor and activator transcription
RA: Rheumatoid arthritis
SLE: Systemic lupus erythematosus
MMP: Matrix metalloproteinase
MDSC: Myeloid-derived suppressor cell
HRCT: High resolution computed tomography
FVC: Forced vital capacity
DLCO: Diffusion capacity for carbon monoxide of the lung
ESSDAI: EULAR Sjögren's syndrome disease activity index
FEV1: Forced expiratory volume in one second
AEIP: Acute exacerbation of interstitial pneumonitis
SAE: Serious advent events
6MWT: 6-Minute walk test

## References

[1] Roca F, Dominique S, Schmidt J (2017). Interstitial lung disease in primary Sjögren's syndrome. Autoimmun Rev.

[2] Palm O, Garen T, Berge Enger T (2013). Clinical pulmonary involvement in primary Sjogren's syndrome: prevalence, quality of life and mortality–a retrospective study based on registry data. Rheumatology (Oxford).

[3] Lee AS, Scofield RH, Hammitt KM (2021). Consensus guidelines for evaluation and management of pulmonary disease in Sjögren's. Chest.

[4] Maher TM, Tudor VA, Saunders P (2022). Rituximab versus intravenous cyclophosphamide in patients with connective tissue disease-associated interstitial lung disease in the UK (RECITAL): a double-blind, double-dummy, randomised, controlled, phase 2b trial. Lancet Respir Med.

[5] Luppi F, Sebastiani M, Silva M (2020). Interstitial lung disease in Sjögren's syndrome: a clinical review. Clin Exp Rheumatol.

[6] Gupta S, Ferrada MA, Hasni SA (2019). Pulmonary manifestations of primary Sjögren's syndrome: underlying immunological mechanisms, clinical presentation, and management. Front Immunol.

[7] Wang S, Liu M, Li X (2022). Canonical and noncanonical regulatory roles for JAK2 in the pathogenesis of rheumatoid arthritis-associated interstitial lung disease and idiopathic pulmonary fibrosis. FASEB J.

[8] You H, Xu D, Zhao J (2020). JAK inhibitors: prospects in connective tissue diseases. Clin Rev Allergy Immunol.

[9] Villarino AV, Kanno Y, O'Shea JJ (2017). Mechanisms and consequences of Jak-STAT signaling in the immune system. Nat Immunol.

[10] Clark JD, Flanagan ME, Telliez JB (2014). Discovery and development of Janus kinase (JAK) inhibitors for inflammatory diseases. J Med Chem.

[11] Mok CC (2019). The Jakinibs in systemic lupus erythematosus: progress and prospects. Expert Opin Investig Drugs.

[12] Kim S, Boehme L, Nel L (2022). Defective STAT5 activation and aberrant expression of BCL6 in naive CD4 T cells enhances follicular Th Cell-like differentiation in patients with granulomatosis with polyangiitis. J Immunol.

[13] Puccetti A, Fiore PF, Pelosi A (2018). Gene expression profiling in Behcet's disease indicates an autoimmune component in the pathogenesis of the disease and opens new avenues for targeted therapy. J Immunol Res.

[14] Schwartz DM, Kanno Y, Villarino A (2017). JAK inhibition as a therapeutic strategy for immune and inflammatory diseases. Nat Rev Drug Discov.

[15] O'Shea JJ, Schwartz DM, Villarino AV (2015). The JAK-STAT pathway: impact on human disease and therapeutic intervention. Annu Rev Med.

[16] Boyle DL, Soma K, Hodge J (2015). The JAK inhibitor tofacitinib suppresses synovial JAK1-STAT signalling in rheumatoid arthritis. Ann Rheum Dis.

[17] Kao L, Chung L, Fiorentino DF (2011). Pathogenesis of dermatomyositis: role of cytokines and interferon. Curr Rheumatol Rep.

[18] Kurtzman DJ, Wright NA, Lin J (2016). Tofacitinib citrate for refractory cutaneous dermatomyositis: an alternative treatment. JAMA Dermatol.

[19] You H, Zhang G, Wang Q (2019). Successful treatment of arthritis and rash with tofacitinib in systemic lupus erythematosus: the experience from a single centre. Ann Rheum Dis.

[20] Liu Y, Ji Z, Yu W (2021). Tofacitinib for the treatment of antineutrophil cytoplasm antibody-associated vasculitis: a pilot study. Ann Rheum Dis.

[21] Lescoat A, Lelong M, Jeljeli M (2020). Combined anti-fibrotic and anti-inflammatory properties of JAK-inhibitors on macrophages in vitro and in vivo: perspectives for scleroderma-associated interstitial lung disease. Biochem Pharmacol.

[22] Aung WW, Wang C, Xibei J (2021). Immunomodulating role of the JAKs inhibitor tofacitinib in a mouse model of bleomycin-induced scleroderma. J Dermatol Sci.

[23] Moriana C, Moulinet T, Jaussaud R (2022). JAK inhibitors and systemic sclerosis: a systematic review of the literature. Autoimmun Rev.

[24] Sendo S, Saegusa J, Yamada H (2019). Tofacitinib facilitates the expansion of myeloid-derived suppressor cells and ameliorates interstitial lung disease in SKG mice. Arthritis Res Ther.

[25] Tardella M, Di Carlo M, Carotti M (2022). A retrospective study of the efficacy of JAK inhibitors or abatacept on rheumatoid arthritis-interstitial lung disease. Inflammopharmacology.

[26] Chen Z, Wang X, Ye S (2019). Tofacitinib in amyopathic dermatomyositis-associated interstitial lung disease. N Engl J Med.

[27] Romero-Bueno F, Diaz Del Campo P, Trallero-Araguás E (2020). Recommendations for the treatment of anti-melanoma differentiation-associated gene 5-positive dermatomyositis-associated rapidly progressive interstitial lung disease. Semin Arthritis Rheum.

[28] Barrera MJ, Aguilera S, Castro I (2021). Tofacitinib counteracts IL-6 overexpression induced by deficient autophagy: implications in Sjögren's syndrome. Rheumatology (Oxford).

[29] Lee J, Lee J, Kwok SK (2018). JAK-1 Inhibition suppresses interferon-induced BAFF production in human salivary gland: potential therapeutic strategy for primary Sjögren's syndrome. Arthritis Rheumatol.

[30] Price E, Bombardieri M, Kivitz A (2022). Safety and efficacy of filgotinib, lanraplenib and tirabrutinib in Sjögren's syndrome: a randomized, phase 2, double-blind, placebo-controlled study. Rheumatology (Oxford).

[31] Vitali C, Bombardieri S, Jonsson R (2002). Classification criteria for Sjögren's syndrome: a revised version of the European criteria proposed by the American-European Consensus Group. Ann Rheum Dis.

[32] Shiboski CH, Shiboski SC, Seror R (2017). 2016 American College of Rheumatology/European League Against Rheumatism Classification Criteria for Primary Sjögren's Syndrome: A Consensus and Data-Driven Methodology Involving Three International Patient Cohorts. Arthritis Rheumatol.

[33] Group of Pulmonary Vascular and Interstitial Diseases Associated with Rheumatic Diseases, Chinese Association of Rheumatology and Immunology Physicians, Chinese Rheumatic Disease Data Center (2018). 2018 Chinese expert-based consensus statement regarding the diagnosis and treatment of interstitial lung disease associated with connective tissue diseases. Chin J Intern Med.

[34] American Thoracic Society (2000). Idiopathic pulmonary fibrosis: diagnosis and treatment. International consensus statement. American Thoracic Society (ATS), and the European Respiratory Society (ERS). Am J Respir Crit Care Med.

