# Supplementary material for: Tofacitinib in the treatment of primary Sjögren’s syndrome-associated interstitial lung disease: study protocol for a prospective, randomized, controlled and open-label trial
Source: BMC Pulm Med. 2023 Nov 25;23:473. doi: 10.1186/s12890-023-02774-0 (PMC10676577; doi:10.1186/s12890-023-02774-0)
Supplement: Supplementary file 2 — Additional file 2. [file 12890_2023_2774_MOESM2_ESM.docx]

**Informed Consent Form**

**Project name:** Tofacitinib in the treatment of primary Sjögren’s syndrome-associated interstitial lung disease: study protocol for a prospective, randomized, controlled and open-label trial

**Version: 1.0**

**Research unit:** Shanghai Tongji Hospital

**Principal investigator:** Xuan Wang

You are being invited to participate in a clinical research study. This informed consent form gives you some information to help you decide whether to participate in this clinical study. Please read it carefully and ask the investigator in charge of the study if you have any questions.

Your participation in this study is voluntary. This study has been reviewed by our Institutional Ethics Review Board.

If you have any questions or concerns about this study, you may contact the study physicians: Xuan Wang at 021-66111086.

If you have questions about the rights of the subjects, you can contact the Ethics Committee of Shanghai Tongji Hospital at 021-66111243 or email: tongjilunli2012@163.com.

A total of 120 subjects were planned to be included in this study, and one hospital participated in this study.

**1.Aim and objective**

- To evaluate the efficacy of tofacitinib in the treatment of pSS-ILD after 52 weeks of administration, which will be noninferior to cyclophosphamide (CYC) with azathioprine (AZA).
- To investigate the suitable population, clinical efficacy indicators, effects on cellular and humoral immunity, and possible clinical side effects of tofacitinib for providing a basis for clinical application in pSS-ILD in the future.

**2.Background**

Primary Sjogren's syndrome (pSS) is a chronic inflammatory autoimmune disease, with impaired salivary and lacrimal glands in the early clinical stage, and multi-system and multi-organ involvement in the later stage, among which pulmonary involvement is the most common, while interstitial lung disease (ILD) is the most common form of lung involvement, accounting for 6-70% in different studies. ILD can cause symptoms such as dyspnea, cough, pulmonary hypertension and sleep difficulties, and thus affect the quality of life of patients. 50% of patients with pSS-ILD mainly present with respiratory symptoms at the first visit. The five-year survival rate of patients with pSS-ILD is 80%, which is an important cause of death in patients with pSS.

At present, the treatment of pSS-ILD is an empirical reference to the treatment of other connective tissue diseases (CTD) complicated with ILD, which requires anti-inflammatory drugs (glucocorticoid), anti-fibrosis drugs (nintedanib, pirfenidone), immunosuppressants (hydroxychloroquine, AZA, CYC), and

immunomodulators. In recent years, studies have pointedout that biologic

agents such as rituximab are a safe and effective alternative to CYC in the

treatment of lung manifestations of CTD, and the combination of anti-fibrosis drugs and immunosuppressants has a favorable effect on the improvement of lung function and overall tolerance of patients. However, exploratory studies with large samples are still needed to confirm this.

The previous observation of the research group found that patients with pSS-ILD had high clinical heterogeneity, some patients had rapid progression of lung lesions, obvious cough and dyspnea, poor treatment response, difficult hormone reduction, repeated infection in the course of the disease. In addition, some patients have long-term stable disease with not obvious symptoms, and the progress of chest CT during years of follow-up is not obvious. The response to treatment and prognosis of patients with pSS-ILD vary greatly among different pathological types, and the five-year mortality rate of patients with UIP is greater than 60%. As the disease progresses, the treatment of ILD becomes more difficult and the prognosis becomes worse. Drugs with both anti-inflammatory and antifibrotic effects will be a new choice for the treatment of pSS -ILD.

Janus kinases (JAKs) are intracellular non-receptor tyrosine kinases, and the dysfunction of JAK-STAT pathway has been associated with a variety of immune disorders. In recent years, JAK inhibitors have shown promising efficacy in improving the clinical signs and symptoms of rheumatoid arthritis (RA) and other inflammatory autoimmune diseases. It is now widely used for the treatment of inflammatory and autoimmune diseases such as RA, inflammatory bowel disease, psoriasis and psoriatic arthritis, and more and more clinical trials are being conducted.

Tofacitinib is the first JAK pathway inhibitor with clear mechanism of action. It is a novel oral protein tyrosine kinase inhibitor. Since the product was first approved in the United States on November 6, 2012, it has been approved in more than 50 countries and regions around the world, including China, Japan, Russia, Australia and Canada. A number of laboratory and clinical studies have suggested that tofacitinib has anti-inflammatory and anti-fibrosis effects at the same time, which is of great help to improve patients' clinical symptoms, reduce mortality and the incidence of adverse events. Tofacitinib has great advantages in the treatment of ILD, especially CTD-ILD.

Different pathologic types of pSS-ILD have different characteristics in treatment, mainly depending on the severity of symptoms and the impact on lung function. For patients with progressive or severe disease, first-line treatment is usually glucocorticoid-based, alone or in combination with immunosuppressive drugs. Depending on the severity of ILD, the usual initial dose of glucocorticoid is 0.5-1 mg/kg prednisone daily. The classic regimen is in combination with immunosuppressive drugs such as CYC, or AZA. In previous clinical trials, we have tried tofacitinib for RA-ILD, pSS-ILD. Previous studies have confirmed that tofacitinib can not only exert anti-inflammatory function to a certain extent, but also anti-fibrosis, improve the symptoms of dyspnea in patients, prevent the fibrosis process of ILD, and reduce the occurrence of high-risk events such as acute exacerbation, repeated infection, hormone dosage, etc. Based on this, we designed a prospective, randomized, controlled, open, single-center clinical trial to evaluate the efficacy and safety of tofacitinib versus the classic treatment drug CYC sequential AZA in the treatment of pSS-ILD. To explore the suitable population, clinical efficacy indexes, effects on cellular immunity and humoral immunity, and possible clinical side effects of tofacitinib, so as to provide evidence for future clinical application in the treatment.

**3. Inclusion and exclusion criteria (who can participate in this study?):**

You may participate in this study if you meet the following requirements.

- 18 years ≤ age ≤ 75 years;
- Patients are eligible for the pSS classification criteria for 2002/2016;
- Patients are eligible for the ILD classification criteria;
- Exertive dyspnea is present and the Mahler dyspnea modified index task grade 2 is achieved;
- Lung function: FVC accounted for ≥45% of predicted values, DLCO ≥30% of predicted values, forced expiratory volume in one second (FEV1) /FVC>65%;
- If glucocorticoids are used at the time of screening, the dose of prednisone acetate is less than 30mg/d (or equivalent amounts of other types);
- Patients who haven’t used immunosuppressive agents (including but not limited to CYC, cyclosporin A (CsA), AZA, tacrolimus (FK-506), methotrexate (MTX), leflunomide, MMF, etc.) or have been stopped for ≥3 months at the time of screening; when hydroxychloroquine (HCQ) was used, the dose of HCQ was stable for 3 months or more;
- Patients who are not using biologics (including but not limited to rituximab, infliximab, adalimumab, etanercept, etc.) at the time of screening or have stopped medication for ≥3 months;
- Women of reproductive age must have a negative urine pregnancy test. From the beginning of the screening period until the last use, both fertile women and men must voluntarily use a recognized effective contraception;
- Able to read, understand and give written informed consent.

You may not participate in this study if you meet the following requirements.

- Patients with acute exacerbation of interstitial pneumonitis (AEIP);
- Respiratory failure suggested by arterial blood gas analysis;
- Other lung lesions other than ILD are evaluated by the following criteria:

a: Patients with moderate to severe pulmonary hypertension which is assessed by rheumatological experts need special treatment;

b: Smoking in the past 6 months or now still a smoker;

c: Patients with other severe clinical manifestations of pulmonary disease, such as pulmonary mass or active pulmonary infection;

d: Patients with severe lung disease other than ILD indicated by lung biopsy, alveolar lavage, or HRCT;

- Severe heart, liver, kidney and other important organs lesions;
- The active infection is aggravated by glucocorticoid and immunosuppressive therapy;
- Positive for hepatitis B virus surface antigen or hepatitis C antibody;
- Pregnant and lactating women, or childbearing age can’t ensure effective contraception;
- Tofacitinib, glucocorticoids, CYC, AZA allergy or intolerance.

**4. Research process (what do you need to do if you participate in this study?):**

All subjects who meet the inclusion criteria and have no exclusion criteria will be randomly divided into 2 groups by random number table method in a ratio of 1:1. You have a half chance to be enrolled in the tofacitinib treatment group and a half chance to be enrolled in the control group. Tofacitinib treatment group is given oral tofacitinib 5mg each time, twice a day, and medium dose prednisone 0.25-0.5mg/kg·d; The control group is given cyclophosphamide, azathioprine and medium doses of prednisone 0.25-0.5mg/kg·d. Observation for 52 weeks, after enrollment, the patients are followed up once at 0, 4, 8, 12, 16, 20, 24, 28, 32, 36, 44 and 52 weeks, and the last visit is 52 weeks. The outcome measures include cough symptom score, the health-related quality of life (HARQoL) score, the Mahler dyspnea index, 6-minute walk test (6MWT), ESSDAI, pulmonary function, HRCT, laboratory routine and immune markers. ILD activity status (including HRCT, pulmonary function, symptoms) will be performed at baseline and 52-week treatments to determine the efficacy of treating ILD.

**5. Risks and discomforts:**

Allergy, dizziness, drowsiness, peripheral edema, upper respiratory tract infection, etc. may occur during the course of a trial. Your sample collection will be conducted strictly in accordance with sterility requirements, and there may be some very small risks associated with sample collection, including transient pain, localized blueness, in a few cases mild dizziness, or extremely rare needle infection.

If the subject experiences any discomfort, new changes in condition, or anything unexpected during the study, whether or not related to the drug/test, the subject's primary care physician should be contacted promptly, and the physician will make a judgment call and provide medical treatment.

**6. Possible benefits (what will this study do for me):**

Both the experimental group and the control group can get effective treatment, which is beneficial to the relief of symptoms. Information from the study will help to obtain important information related to the treatment of Sjogren's syndrome associated with organ damage. Your participation may benefit you and future patients who suffer from the same affliction.

**7. Possible additional costs or burdens (will I have to pay anything to participate in this study?):**

There are no additional expenses for you to participate in this research project. The cost of drug used in this study is covered by the research grant.

**8. Compensation received for participating in the study:**

We will pay your reasonable travel expenses for participation in this study, CNY 50 for each clinical visit.

**9. Medical treatment and compensation for injuries (what to do if you are injured while participating in the study):**

If you suffer an injury during study participation or an adverse event during drug treatment, please contact your study physician and you will receive prompt treatment; any injury that is causally related to the study or the drug used in this trial, the sponsor will bear the medical costs and give you appropriate financial compensation in accordance with the relevant national laws and regulations.

Even if you have signed this informed consent form, you retain all your legal rights and interests.

**10. As a research subject, you have the following responsibilities:**

Provide truthful information about your medical history and current medical condition; tell the study doctor about any discomfort you experience during this study; refrain from taking restricted medications, foods, etc.; and tell the study doctor if you have participated in other studies recently or are currently participating in other studies.

**11. Privacy and confidentiality:**

If you decide to participate in this study, we will make every effort to protect your personal privacy to the extent permitted by law. Any public reporting of the results of this study will not disclose any personal information about you. The physician in charge of the study and other researchers will use your medical information to conduct the study. This information may include your name, address, telephone number, medical history, and information obtained at the time of your study visit. Information that identifies you will not be released to members outside the study team unless your permission is obtained. All study members and study sponsors are required to keep your identity confidential. Your file will be kept in a locked file cabinet and will be accessible only to the researcher. To ensure that the research is conducted in accordance with regulations, members of the government administration or ethics review committee will have access to your personal information at the research unit, as required. When the results of this study are published, no personal information about you will be disclosed.

We will contact you promptly about any meaningful new developments or new medical information related to your health during the study, such as suggesting that you undergo tests to determine such new information. I will also keep you informed of any new information that may affect your choice to continue in the study.

**12. Are there any other treatments available and what are the advantages and disadvantages of the treatments used in this study?**

Standard treatment: cyclophosphamide, azathioprine, and moderate doses of prednisone 0.25-0.5mg/kg·d.

**13. Subject rights:**

Participation in the study is entirely voluntary. You may refuse to participate in the study, or withdraw from the study at any time during the study, and your data will not be included in the study results. None of this will affect your relationship with your physician. Any medical treatment or rights you may have will not be affected by this.

The study physician may terminate your participation in the study if you require other treatment, if you fail to comply with the study plan, if you have a study-related injury, or for any other reason.

**Informed Consent Form Signature Page**

I have read this informed consent form and have discussed and asked questions about this study with my doctor. I have been given a detailed explanation of the purpose of the study, the study process, the possible risks and benefits, and all my questions have been answered, and I understand that participation in this study is voluntary.

I acknowledge that I have had sufficient time to consider this, including the possible risks of participating in the study. I am aware that I can consult with my physician at any time for further information, that I can withdraw from the study at any time without discrimination or reprisal, that my medical treatment and benefits will not be affected by withdrawal from the study, and that it would be in my interest and that of the study as a whole to inform my physician of any changes in my condition and to complete appropriate physical and physical examinations if I withdraw from the study, particularly for treatment reasons. If I need to take any other treatment as a result of a change in my condition, I will seek prior advice from my physician or tell him/her truthfully afterwards.

I am voluntarily participating in this study. I give my consent to the investigator, sponsor, health administration supervisory authority/drug and food regulatory authority, and ethics committee to access my study data.

I will be given a signed and dated copy of the informed consent.

Subject's name: _____________. Agent's name: _____________.

Subject's signature: __________. Agent 's signature: _____________.

Date: _____________. Date: _____________.

(Note: Signature of witness required if subject is illiterate and signature of agent required if subject is incapacitated)

I have accurately informed the subject of this document and he/she has accurately read this informed consent form and certify that the subject has had the opportunity to ask questions. I certify that he/she has given voluntary consent.

Name of Investigator: ________________________

Investigator's signature: _________________________

Date: _________________________
